# Frequency of HLA class I and II in an admixed Brazilian population with psoriasis^؆^

**DOI:** 10.1016/j.abd.2025.501258

**Published:** 2026-01-08

**Authors:** Ana Luisa Sampaio, Bruna Romana-Souza, Haizza Monteiro, Jeane de Souza Nogueira, Danielle Angst Secco, Gilson Costa dos Cantos, Andrea Monte-Alto-Costa, Flavia Cassia, Sueli Carneiro, Luna Azulay-Abulafia, Luis Cristóvão Porto

**Affiliations:** a Medical Science Post Graduate Program, Universidade do Estado do Rio de Janeiro, Rio de Janeiro, RJ, Brazil; b Dermatology Service, Hospital Universitário Pedro Ernesto, Universidade do Estado do Rio de Janeiro, Rio de Janeiro, RJ, Brazil; c Laboratory of Tissue Repair, Universidade do Estado do Rio de Janeiro, Rio de Janeiro, RJ, Brazil; d Tissue Repair and Histocompatibility Technological Core, Universidade do Estado do Rio de Janeiro, Rio de Janeiro, RJ, Brazil; e Laboratory of Metabolomic, Universidade do Estado do Rio de Janeiro, Rio de Janeiro, RJ, Brazil; f Instituto de Dermatologia Prof R. D. Azulay, Santa Casa de Misericórdia do Rio de Janeiro, Rio de Janeiro, RJ, Brazil

**Keywords:** Histocompatibility antigens class I, Histocompatibility antigens class II, HLA antigens, Polymorphism, genetic, Psoriasis

## Abstract

**Background:**

Psoriasis is a chronic, immune-mediated disease with a significant genetic component. The HLA-C*06:02 allele is one of the most strongly associated with the disease, particularly influencing early onset and severity. There are few current data on genetics in a Brazilian population with psoriasis.

**Objective:**

This study aimed to investigate the genetic associations between human leucocyte antigen (HLA) alleles and psoriasis in a Brazilian admixed population.

**Methods:**

The authors conducted HLA class I and II genotyping in 144 patients with psoriasis and compared the results with those of 720 controls. Additionally, the authors calculated the Psoriasis Area and Severity Index (PASI) and recorded whether the patient had current or previous systemic treatment for psoriasis and the age of disease onset.

**Results:**

HLA-B*13:02g, B*15:01g, B*37:01g, B*38:01g, B*57:01g, B*57:02g, B*13:02g, C*01:02g, C*06:02g, C*12:03g, C*18:01g, DRB1*01:02g, DRB1*04:08g and DPB1*04:01g alleles were associated with an increased risk of psoriasis (after the Bonferroni correction factor, only the HLA-C*06:02 remained significant). And HLA-DRB1*15:03g conferred protection against psoriasis after Bonferroni correction. Alleles significantly associated with PASI score < 10 were A*34:02g (p = 0.037) and B*50:01g (p = 0.037), while the allele related to PASI > 10 was DRB1*01:01g (p = 0.049). When comparing the age of disease onset, the following alleles were significantly associated with early onset psoriasis (before 30 years of age): B*44:03g (p = 0.010) and C*07:02g (p = 0.022).

**Study limitations:**

The sample size was small compared with other international publications, and the subgroup of patients with mild disease was less represented; however, the combination of analytical approaches (univariate tests, PCA, and correction for multiple comparisons) reinforces the robustness of the work.

**Conclusion:**

The present findings highlight the genetic complexity of psoriasis in a diverse population and suggest that it may not be directly linked to specific genetic factors. Further research is required to explore the environmental and genetic interactions that contribute to psoriasis pathogenesis.

## Introduction

Psoriasis is a chronic, systemic, immune-mediated disease that involves both innate and adaptive immunity. In Brazil, the prevalence is estimated to be 1.31% (1.15% in females and 1.47% in males), with an average age of 52 years.^1^ The prevalence varies regionally, being higher in the south and southeast, likely due to greater European ancestry and lower ultraviolet radiation exposure than in other areas.

Regarding the genetic background, it is well established that the HLA-C*06:02 allele has the strongest association with psoriasis and plays a key role in immune response against melanocytes by presenting autoantigens such as the peptide ADAMTS-like protein 5,^2^ potentially influencing early onset and severity.^3^ Other HLA alleles, including HLA-C*18:01*, a rare allele in Europeans,*^4^ -C*12:02 and -C*07:04 in Japan,^5^ -C*01 in Asia,^6^ -C*12,^7^ and HLA-B and HLA-A, are associated with psoriasis globally.^2^, ^8^, ^9^ Class II HLA genes are less commonly associated with links to HLA-DPB1*05:01 in China^10^ and HLA-DQα1 amino acid position 53 in a cohort of 9,247 Europeans.^11^

In Brazil’s admixed population, limited previous studies have linked psoriasis to HLA-B*57, -B*27, and -C*06, with HLA-A*02∼C*06∼B*57∼DRB1*07∼DQB1*03 as a common haplotype in patients and HLA-B*07 appears to be protective against psoriasis.^12^ Other studies have examined tumor necrosis factor (TNF) polymorphisms and HLA-C.^13^, ^14^, ^15^, ^16^, ^17^

The group also previously studied single-nucleotide polymorphisms (SNPs) in genes related to the IL17 inflammatory pathway and found no association between SNPs rs361525, rs4819554, and rs33980500 in a Brazilian population.^18^ In this study, the authors compared the frequencies of class I and class II HLA genes between psoriasis patients, categorized based on the severity and age of disease onset, and healthy controls.

## Methods

### Psoriatic individuals

Between 2021 and 2023, patients diagnosed with psoriasis (based on medical history, physical examination and skin biopsy) were recruited from the Dermatology Department at University Hospitals in Rio de Janeiro, Brazil. All participants signed an Informed Consent Form (ICF) to confirm their willingness to participate. Inclusion criteria included age 18–70 years, sex, ethnicity, and disease duration of more than 10 years. Patients with psychiatric disorders, intellectual disabilities, or other visible skin diseases were excluded from the study. The Psoriasis Activity Score Index (PASI) was also completed during consultation.

All participants read and signed an informed consent form for inclusion in the study, which was approved by the Research Ethics Committee (CAAE: 41957320.3.0000.5259).

### Samples

Peripheral blood samples were collected in 4 mL EDTA tubes (purple top) from all participants. The samples were transported to a thermal cooler using industrial ice packs.

### DNA extraction

Genomic DNA was extracted from blood samples using the Biopur Mini Spin Plus Kit (Biopur, Biometrix, Curitiba, Brazil) (Catalog: BP100-50, Batch: 32200739). DNA concentration was measured using a NanoDrop spectrophotometer.

### Patient samples

The authors included previous HLA-typed with medium resolution SSOP *HLA-A, -B, -C, -DRB1 and -DQB1* typed results.^12^ The allelic level (4-digit resolution) was imputed by selecting the most common allele for each MAC code in the Brazilian population.^19^ Samples of 85 patients included in the cohort after 2021 were HLA-typed (*HLA-A, -B, -C, -DRB1, -DQB1 and -DPB1*) using a commercial NGS-based HLA kit (Holotype HLA™ NGS Assay; Omixon Inc., Budapest, Hungary) on an Illumina MiSeq sequencer (Illumina, Inc., San Diego, USA).

### Control samples

Genotypic and allelic frequencies were assessed and compared with those in the general Brazilian population. Controls were randomly selected from the Brazilian Bone Marrow Donor Registry (REDOME) and matched to cases based on sex, self-reported color-race (ethnic descent), and geographic region at a ratio of 5:1. In total, 720 healthy controls were included.

HLA alleles within P or G groups were denoted by a lower case ‘g’.^20^

### Statistical analysis

Allelic frequencies, as well as the Hardy-Weinberg balance test, and Class I haplotypes were determined based on conventional expectation maximization, analyzed for HLA class I and II results, and evaluated for their presence in psoriasis patients and controls using Arlequin 3.5.2.2.^21^ The HLA frequencies and HLA Class I A∼C∼B haplotypes, with counts greater than 4 were compared: a) between patients with psoriasis and the control group, b) PASI score, and c) psoriasis age of onset was determined using the EpiInfo software. The odds ratio (OR) and confidence interval (CI; 5%–95%) were calculated, and p (significance level Fisher test, < 0.05), or analysis of variance (ANOVA) was applied when appropriate, the pC (p correction - Bonferroni factor)

The loading plots were created on the platform MetaboAnalyst 5.0 Website using log_10_ normalized data, auto-scale features, Euclidean distance measure, and Ward clustering method, with the top 10 different alleles selected after the *t*-test results.^22^, ^23^ The authors also sought to identify alleles among psoriatic patients with differences between mild vs. moderate to severe according to the PASI score (Psoriasis was defined as mild if PASI than 10 and moderate to severe if ≥ 10), and/or previous or current use of systemic treatment (classified as moderate to severe disease), and if the disease appeared before or after 30 years of age.

## Results

### HLA alleles in psoriatic patients and controls

The study included 144 patients with psoriasis (84 men and 60 women) and 720 healthy individuals (420 men and 300 women). The mean age of the disease group was 29 ± 15.5 (5–68) years-old and 34% self-declared as *branca* (white-European descent), 13.9% as *preta* (black -sub-Saharan African descent), and 52.1% as *parda* (admixed) in both the case and control groups (Table 1).

**Table 1:** Sex, age, and ethnicity of control subjects and patients with psoriasis, as well as PASI scores and age at disease onset.

	Control (n = 720)	Psoriasis (n = 144)	p-value
**Sex (F/M)**	300/420	60/84	0.498
Age onset	26.5 ± 4.9 [18–47]	29.0 + 15.5 [5–68]	<0.001
**Skin color**			
White	245 (34.0%)	49 (34.0%)	
Black	100 (13.9%)	20 (13.9%)	
Brown	375 (52.1%)	75 (52.1%)	
**Severity of psoriasis**^a^			
Mild		25 (17.4%)	
Moderate to severe		119 (82.5%)	

Demographic and clinical characteristics of control subjects and patients with psoriasis. Data include sex distribution, age at disease onset, skin color, Psoriasis Area and Severity Index (PASI) scores, and disease severity classification. Patients with previous or current high PASI or undergoing systemic treatment were classified as having severe disease.

a Previous or current high PASI; patients with systemic treatment were considered to have severe PASI.

Psoriasis HLA loci pairwise population was significantly different from the control (FST = 0.03485, *p* < 0.05). When considering the Hardy-Weinberg equilibrium of the studied population (Table S1), HLA*-DQB1* was the only locus that was not in equilibrium. Linkage disequilibrium (LD) analysis among HLA loci (Table S2) demonstrated that, in the psoriasis group, most pairs of loci were in linkage disequilibrium, particularly across Class I regions (A∼C, A∼B, C∼B) and between Class I and Class II loci (A∼DRB1, B∼DRB1, C∼DRB1).

The alleles associated with disease protection (in crescent OR order) and risk were presented in Table 2. HLA-DRB1*15:03 g was found to be protective, and C*06:02 g is the strongest risk allele for psoriasis. Allele frequencies in controls and in psoriasis patients are detailed in Tables S3 and S4, for class I (HLA- A, -B and C) and class II (HLA-DRB1, DQB1 and DPB1) alleles, respectively.

**Table 2:** Protective and risk HLA alleles in controls and psoriasis individuals.

Alleles	Control		Psoriasis		OR [5%‒95%]	*p*	pC
	n	%	n	%			
**Protective**							
DRB1*15:03g	83	11.5%	5	3.5%	0.27 (0.11‒0.69)	0.001	**0.027**
B*07:02g	98	13.6%	8	5.6%	0.37 (0.17‒0.78)	0.002	
C*17:01g	51	7.1%	4	2.8%	0.37 (0.13–1.05)	0.032	
B*44:03g	85	11.8%	8	5.6%	0.44 (0.20‒0.92)	0.010	
C*07:02g	121	16.8%	14	9.7%	0.53 (0.29‒0.95)	0.014	
DQB1*06:02g	171	23.8%	22	15.3%	0.57 (0.35‒0.94)	0.011	
**Risk**							
B*57:02g	2	0.3%	3	2.1%	7.63 (1.26–6.13)	0.035	
DRB1*04:08g	5	0.7%	4	2.8%	4.08 (1.08–5.40)	0.047	
B*13:02g	18	2.5%	10	6.9%	2.91 (1.31–6.44)	0.007	
B*38:01g	30	4.2%	15	10.4%	2.67 (1.40–5.11)	0.003	
C*01:02g	19	2.6%	9	6.3%	2.46 (1.09–5.55)	0.021	
B*37:01g	16	2.2%	7	4.9%	2.24 (0.90–5.56)	0.049	
C*06:02g	110	15.3%	40	27.8%	2.13 (1.40–3.23)	0.000	**0.007**
B*57:01g	34	4.7%	13	9.0%	2.00 (1.02–3.89)	0.026	
B*15:01g	26	3.6%	10	6.9%	1.99 (0.93–4.22)	0.043	
C*12:03g	69	9.6%	25	17.4%	1.98 (1.20–3.26)	0.005	
DPB1*04:01g	37	8.22%	48	13.52%	1.75 [1.11–2.75]	0.008	
DRB1*01:02g	58	8.1%	18	12.5%	1.63 (0.93–2.86)	0.049	

Pc, p-value after Bonferroni correction, * - Fisher test.

Protective and Risk HLA alleles are presented in crescent and decrescent order of Odds Ratio, respectively. The number (n) and percentage (Perc) of samples with individuals presenting the alleles in controls and psoriatic patients (psoriasis) are depicted. Odds Ratio and confidence interval 5% and 95% (OR [5%‒95%]) and p-level significance (p) were estimated with ANOVA with Fisher test when n < 5). Bonferroni correction was applied with a factor representing the number of alleles with n > 5 in each locus; only DRB1*15:03 g for protection and C*06:02 g for risk remained significant.

**Fig. 1:**
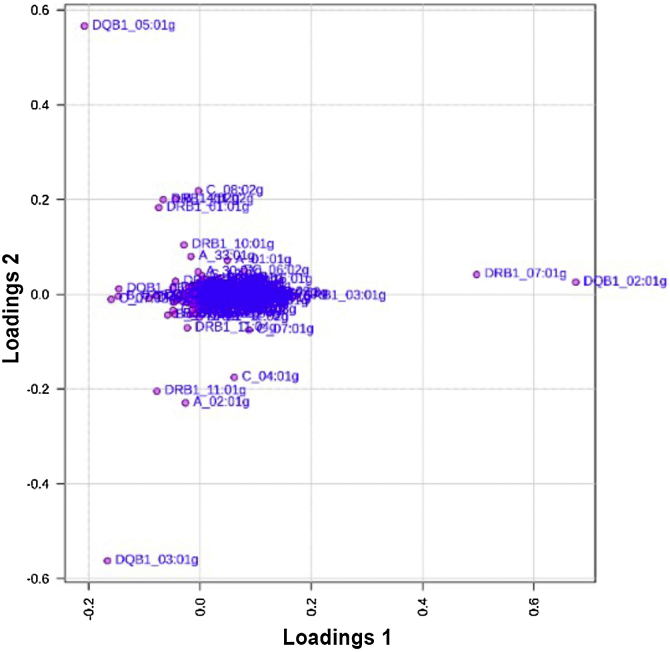
Loading plot of Principal Component Analysis (PCA) applied to HLA alleles comparing psoriasis patients and healthy controls based on HLA frequencies. The loading plot displays the contribution of individual HLA alleles to the first two principal components. Most alleles cluster near the origin, indicating low discriminatory power between groups, while a few alleles ‒ such as HLA-DRB1*07:01 g, HLA-DQB1*02:01 g, and HLA-DQB1*03:01 g ‒ show greater loading distances, suggesting stronger influence in distinguishing psoriasis cases from controls.

The present matrix data included 144 patients and 720 controls with 160 variables: sex, PASI, Onset age, self-reported color, and HLA alleles with a frequency greater than 4 HLA-*A* (n = 29), -*B* (n = 54), -*C* (n = 23), -*DRB1* (n = 35), and -*DQB1* (n = 15). The alleles were scored as 1 (absent), 10 (heterozygosity), or 100 (homozygosity) and log transformed. Principal Component Analysis (PCA) was performed to explore the contribution of HLA alleles to the genetic differentiation between psoriasis patients and healthy controls. The loading plot (Fig. 1) demonstrates that most alleles are clustered near the origin, indicating low discriminative power across groups. In contrast, specific Class II alleles, particularly HLA-DRB1*07:01 g, HLA-DQB1*02:01g, and HLA-DQB1*03:01g, showed higher loading distances, suggesting a stronger influence in distinguishing psoriasis cases from controls.

### Severity, age of onset of psoriasis and HLA alleles

Univariate analysis of the association between HLA alleles and psoriasis severity (Table 3) revealed that A*34:02 g (p = 0.010) and B*50:01 g (p = 0.011) were more frequently observed in patients with mild disease, suggesting a possible protective effect against moderate-to-severe forms. In contrast, the allele DRB1*01:01 g (p = 0.062) was identified exclusively in patients with moderate-to-severe psoriasis, representing a risk marker for greater clinical severity. Regarding age at onset, A*23:01 g (p = 0.006), A*30:01 g (p = 0.003), B*15:03 g (p = 0.078), and DQB1*03:03 (p = 0.026) were associated with late-onset psoriasis (>30 years), whereas B*44:03 g (p = 0.009) and C*07:02 g (p = 0.015) were more frequent in early-onset cases (<30 years), the latter being up to five times more common in this group, thus representing an important marker of risk for type 1 psoriasis (Table 3).

**Table 3:** HLA alleles, severity (PASI) and onset age.

Allele	n	%	n	%	Odds Ratio [5%‒95%]	p
PASI	Mild (<10)		Moderate /Severe (≥10)			
A*34:02g	3	12.0%	2	1.7%	0.13 [0.02‒0.79]	0.037
B*50:01g	3	12.0%	2	1.7%	0.13 [0.02‒0.79]	0.037
DRB1*01:01 g (risk)	0	0.0%	15	12.6%	Undefined	0.049
Onset Age	**>30 y**		**<31 y**			
A*23:01g	10	15.4%	2	2.5%	0.14 [0.03‒0.68]	0.003
A*30:01g	6	9.8%	1	1.2%	0.11 [0.01‒0.95]	0.023
B*15:03g	6	9.8%	1	1.2%	0.11 [0.01‒0.95]	0.023
B*44:03 g	0	0.0%	8	9.6%	Undefined	0.010
C*07:02g	2	3.3%	12	14.5%	4.99 [1.07–23.17]	0.022
DQB1*03:03	9	14.8%	3	3.6%	0.22 [0.06‒0.84]	0.019

HLA-C*06:02, although strongly associated with psoriasis susceptibility, did not show significant differences between severity subgroups (Table 3), corroborating previous findings in the literature.

**Fig. 2:**
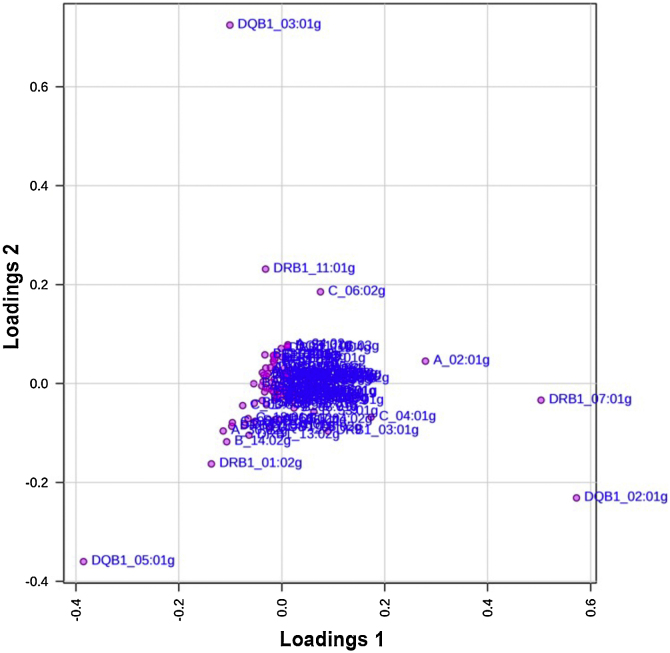
Loading plot from Principal Component Analysis (PCA) comparing psoriasis patients with mild disease (PASI < 10) and those with moderate to severe disease (PASI ≥ 10). The PCA loading plot displays the contribution of HLA alleles to the genetic differentiation between psoriasis subgroups stratified by disease severity. Most alleles are concentrated near the center, indicating limited discriminative impact on clinical severity. However, HLA-C*06:02 g, HLA-DRB1*07:01 g, and HLA-DQB1*02:01 g appear further from the origin, reflecting a stronger contribution to variance and suggesting potential modulation of disease phenotype. The dispersion pattern supports a complex genetic architecture in which certain Class II alleles may influence not only susceptibility but also the clinical expression of psoriasis.

When the Principal Component Analysis (PCA) stratified by disease severity (PASI < 10 versus PASI ≥ 10) was made, it revealed that most HLA alleles clustered centrally, indicating minimal contribution to the differentiation between mild and moderate-to-severe psoriasis (Fig. 2). Nonetheless, HLA-DRB1*07:01g, HLA-DQB1*02:01g, and HLA-C*06:02g, showed greater loading distances, suggesting a potential role in modulating disease phenotype and inflammatory intensity.

### Class I haplotypes in psoriasis individuals and controls, PASI and age onset

Among the 81 *A∼C∼B* haplotypes with count greater than 4, *02:01g∼06:02g∼13:02g* (3.5% × 0.7% OR = 5.14 [1.47–18.91]), *02:01g∼06:02g∼57:01g* (3.5% × 1.0; OR-3.66 [1.15–11.71]) and *26:01g∼12:03g∼38:01g* (4.9% × 1.1% OR = 4,55 [1.62–12.75]) were more frequent among individuals with psoriasis.

## Discussion

This study provides valuable insights into the genetic landscape of psoriasis in an admixed Brazilian population, confirming a significant association between several HLA alleles and disease susceptibility. Consistent with previous research, the authors found that the HLA-C*06:02g allele was one of the strongest genetic markers linked to psoriasis,^2^, ^3^ which aligns with global data on its role in determining the early onset and increased severity of the disease.

Additionally, the authors identified several other HLA alleles, such as HLA-B*15:01g, B*57:01, B*57:02g, B*13:02g, B*38:01g, B*37:01g, C*01:02g, C*18:01g, C*12:03g, DRB1*01:02g and DRB1*04:08g which were associated with an increased risk of psoriasis.

In a previous study conducted in a Pakistani population, the HLA-B*15:01 allele was associated with late-onset psoriasis.^24^ However, no prior study has linked it to stress scores and psoriasis. HLA-B*57 has been identified as a susceptibility allele for psoriasis in both Caucasian^25^ and Asian populations^8^ as well as in previous studies involving the Brazilian population.^12^, ^16^ However, the literature does not specifically mention HLA-B*57:02g in psoriasis.

HLA-B*13:02 has been reported as a susceptibility allele for psoriasis in the Chinese population^8^ and has also been associated with cardiovascular disease.^26^ HLA-B*38:01 has been linked to an increased risk of developing psoriatic arthritis (PsA) among individuals with psoriasis.^27^ Although HLA-B*37:01 has not been well documented in the context of psoriasis, one study has associated its presence with treatment response.^28^ HLA-C*01:02 has been identified as a risk allele for psoriasis in a Chinese population,^29^ whereas HLA-C*18:01 has been associated with difficult-to-treat psoriasis in a Caucasian European population.^4^ The remaining alleles have not previously been reported to be associated with psoriasis.

A previous Brazilian research group identified an association between HLA-DRB1*15 and multiple sclerosis, but not psoriasis^30^ or as a protective factor against the disease.

Interestingly, some alleles, particularly HLA-DRB1*15:03g and HLA-DQB1*02:02g exhibited protective effects against psoriasis in the Brazilian population, further emphasizing the complex link between genetic factors and disease susceptibility. Contrary to previous findings in the literature, where an association between psoriasis and HLA-DQB1*02 was reported in a Slovak population,^31^ the present results indicated that this allele confers protection against the disease.

The DRB1∼DQB1 pair analysis showed loss of LD, suggesting partial recombination or allelic heterogeneity within this segment in the psoriasis population. Such findings may reflect the impact of disease-associated selective forces or the unique genetic admixture of the Brazilian population on HLA haplotype distribution among psoriasis patients.

The HLA-*DQB1* locus deviation from Hardy-Weinberg equilibrium (Table S1) may be consequence of population substructures as allele frequencies may vary between subgroups, specifically in this locus and condition, moreover, in PCA analysis. DQB1 alleles appeared in extreme positions in both Loading 1 and Loading 2. The present results with PCA in loading plots1 and 2 discriminate predominantly class II alleles to differentiate controls for psoriasis patients, after Bonferroni correction, DRB1*15:03g (Table 2), and risk with DQB1*05:01g that also appeared in the loading plots.

Class II alleles are typically not associated with psoriasis. DQB1*02:02 has previously been described as a pharmacogenetic marker and is associated with a better response to acitretin treatment.^32^ In 2005, another Brazilian study also found an association between DRB1*01:02/DQB1*05 (p < 0.05, RR = 5.44) and HLA-DRB1*07:01/DQB1*03 alleles (p < 0.02, RR = 9.00), which correlated with early onset of the disease, as well as an association with the haplotypes HLA-DRB1*01:02/DQB1*05 and HLA-DRB1*07:01/DQB1:03.^33^ DQB1*05:01 is observed in the present study in Fig. 1.

DRB1*07 (OR = 2.56) was previously reported to be associated with psoriasis in the Slovak population, and DQB1*02 (OR = 1.09).^31^ This is also observed (along with DQB1*05:01) in Fig. 1.

In a Chinese Han population, the HLA-DQA1*01:04 and DQA1*02:01 alleles were associated with an increased risk of psoriasis, whereas the HLA-DQA1*05:01 allele was found to have a protective effect against the disease.^34^ However, the present results did not demonstrate the presence of these alleles.

On loading plot (Fig. 1), specific Class II alleles, particularly HLA-DRB1*07:01 g, HLA-DQB1*02:01g, and HLA-DQB1*03:01g, showed higher loading distances, suggesting a stronger influence in distinguishing psoriasis cases from controls. These findings support the notion that, although the majority of alleles contribute minimally to total genetic variance, certain loci may exert disproportionate effects in defining disease susceptibility profiles within the Brazilian population. The alleles such as DRB1*15:03g and DQB1*02:02g, identified as protective, appear in peripheral positions because they contribute to differentiating patients from controls. Classical risk alleles, such as C*06:02g and DRB1*07:01g, are also more distant from the center, confirming their weight in the plot. The central clustering of several alleles shows those with no significant impact on differentiation.

### HLA and severity

HLA-A*34:02g and HLA-B*50:01g had lower frequencies in patients with moderate to severe PASI than in controls, whereas DRB1*01:01g was detected only in cases of moderate to severe PASI. The discrepancy between these results and those in the literature may be due to the smaller sample size of mild disease cases (17.4%) compared to moderate to severe cases (82.5%).

Another Brazilian group found an association of more severe disease in male patients with alleles B*37, C*06, C*12, and DRB1*07, whereas B*57 was associated with mild disease.^14^

A larger study in the Brazilian population with a larger sample size of mild disease cases is warranted for further research.

Nonetheless, on loading plot (Fig. 2), HLA-DRB1*07:01g, HLA-DQB1*02:01g, and HLA-C*06:02g, showed greater loading distances, suggesting a potential role in modulating disease phenotype and inflammatory intensity. These alleles, previously associated with susceptibility in the overall cohort, also appear to influence the genetic structure of patients with more severe clinical forms. The pattern supports the hypothesis that while several loci contribute to disease risk, specific Class I and II alleles may further shape the phenotypic spectrum and therapeutic responsiveness of psoriasis in the Brazilian population.

### HLA and age onset of psoriasis

Univariate analysis was performed and showed that alleles associated with psoriasis onset after 30 years of age (type 2 psoriasis) were: A*23:01g (p = 0.006), A*30:01g (p = 0.003), B*15:03g (p = 0.078), and DQB1*03:03 (p = 0.026), in contrast, B*44:03g (p = 0.009) and C*07:02g (p = 0.015) were detected mainly before 30y and C*07:02g was 5 times more frequent in these young patients (14.5% × 3.3%).

Choonhakarn et al., in a Thai population, identified the alleles HLA-A*01, A*02:07, A*30, B*08, B*13, B*46:01, B*57, C*01, C*06:02 (the strongest association), and DRB1*07 as being associated with type I psoriasis (onset before 30 years of age), while higher frequencies of A*02:07, HLA-A*30, C*01 and DRB1*14:01 were significantly associated with psoriasis onset after 30 years of age.^35^ In a Turkish population, Atasoy et al. found that alleles B*57, HLA-*Cw6*, and DRB1*07 are significantly associated with type I psoriasis.^36^

Kim et al. studied the Korean population and found that the haplotype HLA-A*30-B*13-C*06:02-DRB1*07-DQA1*02-DQB1*02 is a high-risk factor for the disease, particularly at an early age in females.^37^ The haplotype HLA-A*33-B*44-C*14:01-DRB1*13-DQA1*01-DQB1*06-DPB1*04:01 was identified as a protective haplotype for psoriasis, whereas the extended haplotype HLA*-A*1*-B*37-*Cw*0602-DRB1*10-DQA1*01-DQB1*05 was found to be a high-risk factor for psoriasis in Koreans.^37^ The Pakistani population previously discussed had HLA alleles B*57, C*06:02, and DQB1*03:03:02 that were strongly associated with early-onset psoriasis, whereas alleles B*15, DRB1*13:02, and DQB1*03:03:02 were associated with late-onset psoriasis.^24^

This study had some limitations that should be acknowledged. The relatively small sample size, particularly within the mild disease subgroup, may have limited the statistical power to detect subtle genetic associations and affected the generalizability of the findings. Additionally, the cross-sectional design precludes longitudinal assessment of disease progression and long-term outcomes associated with specific HLA alleles. The reliance on the self-reported age of onset introduces potential recall bias, and the use of a single PASI assessment does not account for intra-individual variability over time. However, this limitation was mitigated by incorporating additional variables, such as previous or current use of systemic treatment, to classify patients with moderate to severe disease. Furthermore, given the high degree of admixture in the Brazilian population, undetected population substructures may have influenced allele frequency distributions, contributing to potential confounding factors. Despite these limitations, this study provides important and novel data regarding HLA associations in an admixed Brazilian cohort, a population that is underrepresented in global psoriasis research. The use of comprehensive statistical approaches, including univariate and principal component analyses, enhances the robustness of the findings and underscores the potential of certain HLA alleles to serve as biomarkers for disease susceptibility and severity in this unique population. Future larger, longitudinal studies are warranted to confirm these associations and further explore their clinical implications.

In conclusion, the findings of this study underscore the complexity and diversity of HLA allele associations in psoriasis across different populations. Although several alleles have been consistently linked to psoriasis in various populations, the present study adds to the growing body of evidence suggesting population-specific variations in genetic risk factors. The differences observed between the present results and those reported in other populations highlight the need for a more nuanced understanding of how genetic susceptibility varies across ethnicities.

## ORCID IDs

Bruna Romana-Souza: 0000-0001-5665-8694

Haizza Monteiro: 0000-0001-9032-7792

Danielle Angst Secco: 0000-0002-9514-1871

Gilson Costa dos Santos Jr.: 0000-0002-2038-2267

Andrea Monte-Alto-Costa: 0000-0001-6572-7882

Flavia Cassia: 0000-0001-6944-3000

Sueli Carneiro: 0000-0001-7515-2365

Luna Azulay-Abulafia: 0000-0002-4698-2009

Luis Cristóvão Porto: 0000-0003-1499-1821

## Authors' contributions

Ana Luisa Sampaio: Analysis and interpretation of data; writing of the manuscript or critical review of important intellectual content; final approval of the final version of the manuscript.

Bruna Romana-Souza: Study concept and design; analysis and interpretation of data; critical review of important intellectual content; final approval of the final version of the manuscript.

Haizza Monteiro: Analysis and interpretation of data; final approval of the final version of the manuscript.

Jeane de Souza Nogueira: Data collection, analysis and interpretation; critical review of important intellectual content; final approval of the final version of the manuscript.

Danielle Angst Secco: Data collection, analysis and interpretation; critical review of important intellectual content; final approval of the final version of the manuscript.

Gilson Costa dos Santos Jr.: Statistical analysis; final approval of the final version of the manuscript.

Andrea Monte-Alto-Costa: Study concept and design; critical review of important intellectual content; final approval of the final version of the manuscript.

Flavia Cassia: Critical review of important intellectual content; final approval of the final version of the manuscript.

Sueli Carneiro: Study concept and design; critical review of important intellectual content; final approval of the final version of the manuscript.

Luna Azulay-Abulafia: Study concept and design; effective participation in the research guidance; critical review of important intellectual content; final approval of the final version of the manuscript.

Luis Cristóvão Porto: Study concept and design; effective participation in the research guidance; statistical analysis; critical review of important intellectual content; final approval of the final version of the manuscript.

## Financial support

This work was supported by 10.13039/501100004586FAPERJ (Fundação de Amparo à Pesquisa do Estado do Rio de Janeiro – grant E-26/202.683/2019) and 10.13039/501100003593CNPq (Conselho Nacional de Desenvolvimento Científico e Tecnológico – grant 310885/2022-0). 10.13039/501100002322CAPES (Coordenação de Aperfeiçoamento de Pessoal de Nível Superior) funded Ana Luisa Sampaio’s PhD scholarship.

## Research data availability

The entire dataset supporting the results of this study was published in this article.

## Conflicts of interest

None declared.
