# Supplementary material for: Frequency of HLA class I and II in an admixed Brazilian population with psoriasis
Source: An Bras Dermatol. 2026 Jan 8;101(1):501258. doi: 10.1016/j.abd.2025.501258 (PMC12813529; doi:10.1016/j.abd.2025.501258)
Supplement: Supplementary file 1 [file mmc1.docx]

**ABD-D-25-00555_Supplementary Material**

**Table S1** Hardy-Weinberg equilibrium of HLA *loci*.

|  | **Control (n = 720)** | | | **Psoriasis (n = -144)** | | |
| --- | --- | --- | --- | --- | --- | --- |
| ***Locus*** | **Obs. Het.** | **Exp. Het.** | **p-value** | **Obs. Het.** | **Exp. Het.** | **p-value** |
| *A* | 0.90694 | 0.92330 | 0.157 | 0.87500 | 0.91137 | 0.330 |
| *C* | 0.95833 | 0.96765 | 0.688 | 0.97222 | 0.97367 | 0.678 |
| *B* | 0.91806 | 0.92622 | 0.670 | 0.91667 | 0.92039 | 0.986 |
| *DRB1* | 0.95417 | 0.94582 | 0.753 | 0.90972 | 0.94304 | 0.746 |
| *DQB1* | 0.87222 | 0.87260 | 0.499 | 0.77083 | 0.87256 | 0.005 |

Obs. Het., Observed Heterogeneity; Exp. Het., Expected heterogeneity.

**Table S2** Linkage Disequilibrium between locus pairs in the psoriasis sample.

| **Locus** | **A** | **C** | **B** | **DRB1** | **DQB1** |
| --- | --- | --- | --- | --- | --- |
| **A** | ***** | **+** | **+** | **+** | **+** |
| **C** | **+** | ***** | **+** | **+** | **+** |
| **B** | **+** | **+** | ***** | **+** | **+** |
| **DRB1** | **+** | **+** | **+** | ***** | **+** |
| **DQB1** | **+** | **-** | **+** | **+** | ***** |

**Table S3** Class I alleles and frequencies.

|  | **Control** | **Psoriaisis** | **Total** |  | **Control** | **Psoriaisis** | **Total** |  | **Control** | **Psoriaisis** | **Total** |
| --- | --- | --- | --- | --- | --- | --- | --- | --- | --- | --- | --- |
| ***A*** | **n (%)** | **n (%)** | **n (%)** | ***B*** | **n (%)** | **n (%)** | **n (%)** | ***C*** | **n (%)** | **n (%)** | **n (%)** |
| ***01:01g*** | 112 (7.78) | 21 (7.29) | 133 (7.7) | ***07:02g*** | 102 (7.08) | 8 (2.78) | 110 (6.37) | ***01:02g*** | 19 (1.32) | 9 (3.13) | 28 (1.62) |
| ***01:02g*** | 2 (0.14) | 2 (0.69) | 4 (0.23) | ***07:05g*** | 4 (0.28) | 0 (0) | 4 (0.23) | ***01:22*** | 1 (0.07) | 0 (0) | 1 (0.06) |
| ***01:03g*** | 1 (0.07) | 0 (0) | 1 (0.06) | ***08:01g*** | 59 (4.1) | 7 (2.43) | 66 (3.82) | ***01:273*** | 0 (0) | 1 (0.35) | 1 (0.06) |
| ***02:01g*** | 287 (19.9) | 68 (23.6) | 355 (20.5) | ***13:01g*** | 0 (0) | 1 (0.35) | 1 (0.06) | ***02:02g*** | 59 (4.1) | 11 (3.82) | 70 (4.05) |
| ***02:02g*** | 32 (2.22) | 2 (0.69) | 34 (1.97) | ***13:02g*** | 18 (1.25) | 11 (3.82) | 29 (1.68) | ***02:10g*** | 46 (3.19) | 5 (1.74) | 51 (2.95) |
| ***02:04g*** | 4 (0.28) | 0 (0) | 4 (0.23) | ***14:01g*** | 28 (1.94) | 2 (0.69) | 30 (1.74) | ***03:02g*** | 8 (0.56) | 3 (1.04) | 11 (0.64) |
| ***02:05g*** | 14 (0.97) | 2 (0.69) | 16 (0.93) | ***14:02g*** | 70 (4.86) | 19 (6.6) | 89 (5.15) | ***03:03g*** | 32 (2.22) | 8 (2.78) | 40 (2.31) |
| ***02:11g*** | 8 (0.56) | 3 (1.04) | 11 (0.64) | ***14:03*** | 1 (0.07) | 0 (0) | 1 (0.06) | ***03:04g*** | 85 (5.90) | 15 (5.21) | 99 (5.73) |
| ***02:12*** | 1 (0.07) | 0 (0) | 1 (0.06) | ***15:01g*** | 26 (1.81) | 10 (3.47) | 36 (2.08) | ***04:01g*** | 246 (17.0) | 45 (15.6) | 291 (16.8) |
| ***02:19*** | 0 (0) | 1 (0.35) | 1 (0.06) | ***15:03g*** | 42 (2.92) | 7 (2.43) | 49 (2.84) | ***04:07g*** | 1 (0.07) | 0 (0) | 1 (0.06) |
| ***02:20g*** | 2 (0.14) | 0 (0) | 2 (0.12) | ***15:04g*** | 3 (0.21) | 0 (0) | 3 (0.17) | ***05:01g*** | 79 (5.48) | 11 (3.82) | 90 (5.15) |
| ***02:45*** | 1 (0.07) | 0 (0) | 1 (0.06) | ***15:05g*** | 0 (0) | 2 (0.69) | 2 (0.12) | ***06:02g*** | 114 (7.92) | 42 (14.5) | 156 (9.03) |
| ***03:01g*** | 124 (8.61) | 23 (7.99) | 147 (8.51) | ***15:08g*** | 4 (0.28) | 0 (0) | 4 (0.23) | ***07:01g*** | 132 (9.17) | 31 (10.7) | 163 (9.43) |
| ***03:02g*** | 1 (0.07) | 0 (0) | 1 (0.06) | ***15:09g*** | 0 (0) | 1 (0.35) | 1 (0.06) | ***07:02g*** | 127 (8.82) | 14 (4.86) | 140 (8.1) |
| ***11:01g*** | 61 (4.24) | 17 (5.9) | 78 (4.51) | ***15:10g*** | 34 (2.36) | 5 (1.74) | 39 (2.26) | ***07:04g*** | 17 (1.18) | 5 (1.74) | 22 (1.27) |
| ***23:01g*** | 97 (6.74) | 12 (4.17) | 109 (6.31) | ***15:123g*** | 1 (0.07) | 0 (0) | 1 (0.06) | ***07:05*** | 1 (0.07) | 0 (0) | 1 (0.06) |
| ***23:05*** | 3 (0.21) | 0 (0) | 3 (0.17) | ***15:16g*** | 10 (0.69) | 2 (0.69) | 12 (0.69) | ***08:01g*** | 2 (0.14) | 1 (0.35) | 3 (0.17) |
| ***24:02g*** | 105 (7.29) | 27 (9.38) | 132 (7.64) | ***15:17g*** | 9 (0.63) | 3 (1.04) | 12 (0.69) | ***08:02g*** | 93 (6.46) | 19 (6.6) | 112 (6.48) |
| ***24:03g*** | 12 (0.83) | 1 (0.35) | 13 (0.75) | ***15:18g*** | 3 (0.21) | 0 (0) | 3 (0.17) | ***08:03g*** | 1 (0.07) | 0 (0) | 1 (0.06) |
| ***25:01g*** | 17 (1.18) | 4 (1.39) | 21 (1.22) | ***15:20g*** | 3 (0.21) | 0 (0) | 3 (0.17) | ***08:04g*** | 6 (0.42) | 1 (0.35) | 7 (0.41) |
| ***26:01g*** | 43 (2.99) | 11 (3.82) | 54 (3.13) | ***15:31g*** | 1 (0.07) | 0 (0) | 1 (0.06) | ***12:02g*** | 10 (0.69) | 3 (1.04) | 13 (0.75) |
| ***26:08g*** | 1 (0.07) | 1 (0.35) | 2 (0.12) | ***15:37*** | 0 (0) | 1 (0.35) | 1 (0.06) | ***12:03g*** | 70 (4.86) | 25 (8.68) | 93 (5.38) |
| ***26:27*** | 1 (0.07) | 0 (0) | 1 (0.06) | ***15:47g*** | 1 (0.07) | 0 (0) | 1 (0.06) | ***12:12*** | 0 (0) | 1 (0.35) | 1 (0.06) |
| ***26:31*** | 1 (0.07) | 0 (0) | 1 (0.06) | ***18:01g*** | 47 (3.26) | 9 (3.13) | 56 (3.24) | ***14:02g*** | 34 (2.36) | 6 (2.08) | 40 (2.31) |
| ***29:01g*** | 1 (0.07) | 0 (0) | 1 (0.06) | ***18:04g*** | 0 (0) | 1 (0.35) | 1 (0.06) | ***14:03g*** | 1 (0.07) | 0 (0) | 1 (0.06) |
| ***29:02g*** | 58 (4.03) | 7 (2.43) | 65 (3.76) | ***18:05g*** | 3 (0.21) | 0 (0) | 3 (0.17) | ***15:02g*** | 47 (3.26) | 4 (1.39) | 51 (2.95) |
| ***30:01g*** | 57 (3.96) | 7 (2.43) | 64 (3.7) | ***27:02g*** | 3 (0.21) | 0 (0) | 3 (0.17) | ***15:03*** | 1 (0.07) | 0 (0) | 1 (0.06) |
| ***30:02g*** | 50 (3.47) | 11 (3.82) | 61 (3.53) | ***27:03*** | 1 (0.07) | 1 (0.35) | 2 (0.12) | ***15:04g*** | 1 (0.07) | 0 (0) | 1 (0.06) |
| ***30:04g*** | 6 (0.42) | 2 (0.69) | 8 (0.46) | ***27:05g*** | 20 (1.39) | 3 (1.04) | 23 (1.33) | ***15:05g*** | 13 (0.83) | 2 (0.69) | 13 (0.75) |
| ***30:07*** | 1 (0.07) | 0 (0) | 1 (0.06) | ***27:12g*** | 1 (0.07) | 0 (0) | 1 (0.06) | ***15:08g*** | 1 (0.07) | 0 (0) | 1 (0.06) |
| ***31:01g*** | 57 (3.96) | 7 (2.43) | 64 (3.7) | ***35:01g*** | 67 (4.65) | 13 (4.51) | 80 (4.63) | ***15:24*** | 1 (0.07) | 0 (0) | 1 (0.06) |
| ***31:02g*** | 1 (0.07) | 0 (0) | 1 (0.06) | ***35:02g*** | 25 (1.74) | 5 (1.74) | 30 (1.74) | ***16:01g*** | 72 (5) | 11 (3.82) | 83 (4.8) |
| ***31:04g*** | 1 (0.07) | 0 (0) | 1 (0.06) | ***35:03g*** | 28 (1.94) | 8 (2.78) | 36 (2.08) | ***16:02g*** | 11 (0.76) | 3 (1.04) | 14 (0.81) |
| ***32:01g*** | 32 (2.22) | 5 (1.74) | 37 (2.14) | ***35:04g*** | 6 (0.42) | 2 (0.69) | 8 (0.46) | ***16:04g*** | 1 (0.07) | 0 (0) | 1 (0.06) |
| ***33:01g*** | 47 (3.26) | 9 (3.13) | 56 (3.24) | ***35:05g*** | 11 (0.76) | 1 (0.35) | 12 (0.69) | ***17:01g*** | 53 (3.68) | 4 (1.39) | 57 (3.3) |
| ***33:03g*** | 23 (1.6) | 4 (1.39) | 27 (1.56) | ***35:08g*** | 12 (0.83) | 4 (1.39) | 16 (0.93) | ***18:01g*** | 28 (1.94) | 7 (2.43) | 25 (2.12) |
| ***33:05*** | 1 (0.07) | 0 (0) | 1 (0.06) | ***35:11g*** | 3 (0.21) | 0 (0) | 3 (0.17) |  |  |  |  |
| ***34:02g*** | 20 (1.39) | 5 (1.74) | 25 (1.45) | ***35:20g*** | 3 (0.21) | 0 (0) | 3 (0.17) |  |  |  |  |
| ***36:01g*** | 12 (0.83) | 3 (1.04) | 15 (0.87) | ***35:43g*** | 0 (0) | 1 (0.35) | 1 (0.06) |  |  |  |  |
| ***36:04*** | 0 (0) | 1 (0.35) | 1 (0.06) | ***37:01g*** | 16 (1.11) | 7 (2.43) | 23 (1.33) |  |  |  |  |
| ***66:01g*** | 10 (0.69) | 1 (0.35) | 11 (0.64) | ***38:01g*** | 30 (2.08) | 15 (5.21) | 45 (2.6) |  |  |  |  |
| ***66:02g*** | 5 (0.35) | 1 (0.35) | 6 (0.35) | ***38:02g*** | 1 (0.07) | 0 (0) | 1 (0.06) |  |  |  |  |
| ***66:03g*** | 1 (0.07) | 0 (0) | 1 (0.06) | ***39:01g*** | 14 (0.97) | 2 (0.69) | 16 (0.93) |  |  |  |  |
| ***68:01g*** | 54 (3.75) | 13 (4.51) | 67 (3.88) | ***39:03g*** | 2 (0.14) | 0 (0) | 2 (0.12) |  |  |  |  |
| ***68:02g*** | 45 (3.13) | 8 (2.78) | 53 (3.07) | ***39:05g*** | 9 (0.63) | 3 (1.04) | 12 (0.69) |  |  |  |  |
| ***68:27g*** | 1 (0.07) | 0 (0) | 1 (0.06) | ***39:06g*** | 4 (0.28) | 2 (0.69) | 6 (0.35) |  |  |  |  |
| ***68:67*** | 1 (0.07) | 0 (0) | 1 (0.06) | ***39:09g*** | 3 (0.21) | 0 (0) | 3 (0.17) |  |  |  |  |
| ***69:01g*** | 0 (0) | 2 (0.69) | 2 (0.12) | ***39:10g*** | 5 (0.35) | 0 (0) | 5 (0.29) |  |  |  |  |
| ***74:01g*** | 23 (1.6) | 5 (1.74) | 28 (1.62) | ***39:13g*** | 4 (0.28) | 0 (0) | 4 (0.23) |  |  |  |  |
| ***80:01g*** | 3 (0.21) | 2 (0.69) | 5 (0.29) | ***39:24g*** | 2 (0.14) | 0 (0) | 2 (0.12) |  |  |  |  |
|  |  |  |  | ***40:01g*** | 24 (1.67) | 4 (1.39) | 28 (1.62) |  |  |  |  |
|  |  |  |  | ***40:02g*** | 23 (1.6) | 4 (1.39) | 27 (1.56) |  |  |  |  |
|  |  |  |  | ***40:04g*** | 8 (0.56) | 3 (1.04) | 11 (0.64) |  |  |  |  |
|  |  |  |  | ***40:06g*** | 2 (0.14) | 1 (0.35) | 3 (0.17) |  |  |  |  |
|  |  |  |  | ***40:09*** | 0 (0) | 1 (0.35) | 1 (0.06) |  |  |  |  |
|  |  |  |  | ***40:16g*** | 1 (0.07) | 0 (0) | 1 (0.06) |  |  |  |  |
|  |  |  |  | ***41:01g*** | 10 (0.69) | 0 (0) | 10 (0.58) |  |  |  |  |
|  |  |  |  | ***41:02g*** | 9 (0.63) | 0 (0) | 9 (0.52) |  |  |  |  |
|  |  |  |  | ***42:01g*** | 31 (2.15) | 3 (1.04) | 34 (1.97) |  |  |  |  |
|  |  |  |  | ***42:02g*** | 7 (0.49) | 0 (0) | 7 (0.41) |  |  |  |  |
|  |  |  |  | ***44:02g*** | 68 (4.72) | 8 (2.78) | 76 (4.4) |  |  |  |  |
|  |  |  |  | ***44:03g*** | 89 (6.18) | 8 (2.78) | 97 (5.61) |  |  |  |  |
|  |  |  |  | ***44:05g*** | 1 (0.07) | 1 (0.35) | 2 (0.12) |  |  |  |  |
|  |  |  |  | ***45:01g*** | 33 (2.29) | 3 (1.04) | 36 (2.08) |  |  |  |  |
|  |  |  |  | ***47:01g*** | 2 (0.14) | 1 (0.35) | 3 (0.17) |  |  |  |  |
|  |  |  |  | ***48:01g*** | 3 (0.21) | 0 (0) | 3 (0.17) |  |  |  |  |
|  |  |  |  | ***48:02g*** | 6 (0.42) | 3 (1.04) | 9 (0.52) |  |  |  |  |
|  |  |  |  | ***48:03g*** | 1 (0.07) | 0 (0) | 1 (0.06) |  |  |  |  |
|  |  |  |  | ***49:01g*** | 32 (2.22) | 9 (3.13) | 41 (2.37) |  |  |  |  |
|  |  |  |  | ***50:01g*** | 35 (2.43) | 5 (1.74) | 40 (2.31) |  |  |  |  |
|  |  |  |  | ***50:02g*** | 2 (0.14) | 0 (0) | 2 (0.12) |  |  |  |  |
|  |  |  |  | ***51:01g*** | 98 (6.81) | 13 (4.51) | 111 (6.42) |  |  |  |  |
|  |  |  |  | ***51:02g*** | 1 (0.07) | 0 (0) | 1 (0.06) |  |  |  |  |
|  |  |  |  | ***51:04g*** | 2 (0.14) | 0 (0) | 2 (0.12) |  |  |  |  |
|  |  |  |  | ***51:07g*** | 2 (0.14) | 1 (0.35) | 3 (0.17) |  |  |  |  |
|  |  |  |  | ***51:08g*** | 3 (0.21) | 0 (0) | 3 (0.17) |  |  |  |  |
|  |  |  |  | ***52:01g*** | 24 (1.67) | 8 (2.78) | 32 (1.85) |  |  |  |  |
|  |  |  |  | ***53:01g*** | 56 (3.89) | 11 (3.82) | 67 (3.88) |  |  |  |  |
|  |  |  |  | ***55:01g*** | 7 (0.49) | 3 (1.04) | 10 (0.58) |  |  |  |  |
|  |  |  |  | ***56:01g*** | 3 (0.21) | 2 (0.69) | 5 (0.29) |  |  |  |  |
|  |  |  |  | ***57:01g*** | 34 (2.36) | 13 (4.51) | 47 (2.72) |  |  |  |  |
|  |  |  |  | ***57:02g*** | 2 (0.14) | 3 (1.04) | 5 (0.29) |  |  |  |  |
|  |  |  |  | ***57:03g*** | 16 (1.11) | 6 (2.08) | 22 (1.27) |  |  |  |  |
|  |  |  |  | ***58:01g*** | 26 (1.81) | 9 (3.13) | 35 (2.03) |  |  |  |  |
|  |  |  |  | ***58:02g*** | 16 (1.11) | 6 (2.08) | 22 (1.27) |  |  |  |  |
|  |  |  |  | ***67:01g*** | 0 (0) | 1 (0.35) | 1 (0.06) |  |  |  |  |
|  |  |  |  | ***73:01g*** | 2 (0.14) | 0 (0) | 2 (0.12) |  |  |  |  |
|  |  |  |  | ***78:01g*** | 3 (0.21) | 1 (0.35) | 4 (0.23) |  |  |  |  |
|  |  |  |  | ***81:01g*** | 18 (1.25) | 1 (0.35) | 19 (1.1) |  |  |  |  |
|  |  |  |  | ***82:01g*** | 1 (0.07) | 0 (0) | 1 (0.06) | **Total** | 1440 | 288 |  |
| **Total** | **1440** | **288** |  | **Total** | 1440 | 288 |  |  |  |  |  |

**Table S4** Class II alleles and frequencies.

|  | **Control** | **Psoriaisis** | **Total** |  | **Control** | **Psoriaisis** | **Total** |  | **Control** | **Psoriaisis** | **Total** |
| --- | --- | --- | --- | --- | --- | --- | --- | --- | --- | --- | --- |
| ***DRB1*** | **n (%)** | **n (%)** | **n (%)** | ***DQB1*** | **n (%)** | **n (%)** | **n (%)** | ***DPB1*** | **n (%)** | **n (%)** | **n (%)** |
| ***01:01g*** | 74 (5.14) | 15 (5.21) | 89 (5.15) | ***02:01g*** | 302 (20.97) | 54 (18.75) | 356 (20.60) | ***01:01g*** | 99 (11.6) | 17 (10) | 116 (11.3) |
| ***01:02g*** | 59 (4.1) | 18 (6.25) | 77 (4.46) | ***02:03g*** | 1 (0.07) | 0 (0) | 1 (0.06) | ***02:01g*** | 133 (15.6) | 19 (11.1) | 152 (14.9) |
| ***01:03g*** | 10 (0.69) | 2 (0.69) | 12 (0.69) | ***03:01g*** | 245 (17.94) | 57 (19.7) | 302 (17.42) | ***02:02g*** | 5 (0.59) | 0 (0) | 5 (0.49) |
| ***03:01g*** | 104 (7.22) | 16 (5.56) | 120 (6.94) | ***03:02g*** | 117 (8.13) | 21 (7.29) | 138 (7.99) | ***03:01g*** | 73 (8.59) | 12 (7.06) | 85 (8.33) |
| ***03:02g*** | 24 (1.67) | 4 (1.39) | 28 (1.62) | ***03:03g*** | 50 (3.47) | 14 (4.86) | 64 (3.7) | ***03:02g*** | 0 (0) | 1 (0.59) | 1 (0.1) |
| ***03:06*** | 0 (0) | 1 (0.35) | 1 (0.06) | ***03:04g*** | 4 (0.28) | 0 (0) | 4 (0.23) | ***04:01g*** | 198 (23.2) | 58 (34.1) | 256 (25.1) |
| ***03:15g*** | 1 (0.07) | 0 (0) | 1 (0.06) | ***03:10g*** | 7 (0.49) | 0 (0) | 7 (0.41) | ***04:02g*** | 132 (15.5) | 26 (15.2) | 158 (15.4) |
| ***04:01g*** | 24 (1.67) | 4 (1.39) | 28 (1.62) | ***03:106*** | 0 (0) | 1 (0.35) | 1 (0.06) | ***05:01g*** | 16 (1.88) | 5 (2.94) | 21 (2.06) |
| ***04:02g*** | 22 (1.53) | 7 (2.43) | 29 (1.68) | ***04:02g*** | 78 (5.42) | 14 (4.86) | 92 (5.32) | ***06:01g*** | 9 (1.06) | 3 (1.76) | 12 (1.18) |
| ***04:03g*** | 14 (0.97) | 5 (1.74) | 19 (1.1) | ***05:01g*** | 215 (14.9) | 52 (18.0) | 267 (15.4) | ***09:01g*** | 2 (0.24) | 1 (0.59) | 3 (0.29) |
| ***04:04g*** | 34 (2.36) | 5 (1.74) | 39 (2.26) | ***05:02g*** | 24 (1.67) | 5 (1.74) | 29 (1.68) | ***10:01g*** | 23 (2.71) | 1 (0.59) | 24 (2.35) |
| ***04:05g*** | 30 (2.08) | 3 (1.04) | 33 (1.91) | ***05:03g*** | 34 (2.36) | 10 (3.47) | 44 (2.55) | ***11:01g*** | 16 (1.88) | 4 (2.35) | 20 (1.96) |
| ***04:06g*** | 1 (0.07) | 0 (0) | 1 (0.06) | ***06:01g*** | 7 (0.49) | 2 (0.69) | 9 (0.52) | ***13:01g*** | 36 (4.24) | 4 (2.35) | 40 (3.92) |
| ***04:07g*** | 10 (0.69) | 0 (0) | 10 (0.58) | ***06:02g*** | 179 (12.4) | 23 (7.99) | 202 (11.6) | ***14:01g*** | 26 (3.06) | 5 (2.94) | 31 (3.04) |
| ***04:08g*** | 5 (0.35) | 4 (1.39) | 9 (0.52) | ***06:03g*** | 98 (6.81) | 16 (5.56) | 114 (6.6) | ***15:01g*** | 7 (0.82) | 1 (0.59) | 8 (0.78) |
| ***04:11g*** | 20 (1.39) | 6 (2.08) | 26 (1.5) | ***06:04g*** | 50 (3.47) | 13 (4.51) | 63 (3.65) | ***16:01g*** | 4 (0.47) | 2 (1.18) | 6 (0.59) |
| ***07:01g*** | 197 (13.6) | 48 (16.6) | 245 (14.1) | ***06:08g*** | 2 (0.14) | 0 (0) | 2 (0.12) | ***17:01g*** | 41 (4.82) | 9 (5.29) | 50 (4.9) |
| ***08:01g*** | 25 (1.74) | 6 (2.08) | 31 (1.79) | ***06:09g*** | 26 (1.81) | 6 (2.08) | 32 (1.85) | ***18:01g*** | 13 (1.53) | 1 (0.59) | 14 (1.37) |
| ***08:02g*** | 7 (0.49) | 1 (0.35) | 8 (0.46) | ***06:11g*** | 1 (0.07) | 0 (0) | 1 (0.06) | ***19:01g*** | 5 (0.59) | 0 (0) | 5 (0.49) |
| ***08:03g*** | 6 (0.42) | 2 (0.69) | 8 (0.46) |  |  |  |  | ***20:01g*** | 2 (0.24) | 0 (0) | 2 (0.2) |
| ***08:04g*** | 30 (2.08) | 3 (1.04) | 33 (1.91) |  |  |  |  | ***23:01g*** | 1 (0.12) | 0 (0) | 1 (0.1) |
| ***08:06g*** | 4 (0.28) | 1 (0.35) | 5 (0.29) |  |  |  |  | ***26:01g*** | 1 (0.12) | 1 (0.59) | 2 (0.2) |
| ***08:07*** | 11 (0.76) | 2 (0.69) | 13 (0.75) |  |  |  |  | ***27:01*** | 2 (0.24) | 0 (0) | 2 (0.2) |
| ***09:01g*** | 33 (2.29) | 8 (2.78) | 41 (2.37) |  |  |  |  | ***28:01g*** | 1 (0.12) | 0 (0) | 1 (0.1) |
| ***10:01g*** | 38 (2.64) | 11 (3.82) | 49 (2.84) |  |  |  |  | ***29:01g*** | 1 (0.12) | 0 (0) | 1 (0.1) |
| ***11:01g*** | 101 (7.01) | 21 (7.29) | 122 (7.06) |  |  |  |  | ***39:01g*** | 3 (0.35) | 0 (0) | 3 (0.29) |
| ***11:02g*** | 30 (2.08) | 3 (1.04) | 33 (1.91) |  |  |  |  | ***85:01g*** | 1 (0.12) | 0 (0) | 1 (0.1) |
| ***11:03g*** | 4 (0.28) | 0 (0) | 4 (0.23) |  |  |  |  |  |  |  |  |
| ***11:04g*** | 32 (2.22) | 5 (1.74) | 37 (2.14) |  |  |  |  |  |  |  |  |
| ***12:01g*** | 20 (1.39) | 5 (1.74) | 25 (1.45) |  |  |  |  |  |  |  |  |
| ***12:02g*** | 2 (0.14) | 2 (0.69) | 4 (0.23) |  |  |  |  |  |  |  |  |
| ***13:01g*** | 115 (7.99) | 18 (6.25) | 133 (7.7) |  |  |  |  |  |  |  |  |
| ***13:02g*** | 79 (5.49) | 18 (6.25) | 97 (5.61) |  |  |  |  |  |  |  |  |
| ***13:03g*** | 27 (1.88) | 4 (1.39) | 31 (1.79) |  |  |  |  |  |  |  |  |
| ***13:05g*** | 3 (0.21) | 1 (0.35) | 4 (0.23) |  |  |  |  |  |  |  |  |
| ***13:16g*** | 1 (0.07) | 1 (0.35) | 2 (0.12) |  |  |  |  |  |  |  |  |
| ***14:01g*** | 34 (2.36) | 10 (3.47) | 44 (2.55) |  |  |  |  |  |  |  |  |
| ***14:02g*** | 11 (0.76) | 2 (0.69) | 13 (0.75) |  |  |  |  |  |  |  |  |
| ***14:04g*** | 2 (0.14) | 2 (0.69) | 4 (0.23) |  |  |  |  |  |  |  |  |
| ***14:06g*** | 1 (0.07) | 1 (0.35) | 2 (0.12) |  |  |  |  |  |  |  |  |
| ***14:new*** | 1 (0.07) | 0 (0) | 1 (0.06) |  |  |  |  |  |  |  |  |
| ***15:01g*** | 68 (4.72) | 11 (3.82) | 79 (4.57) |  |  |  |  |  |  |  |  |
| ***15:02g*** | 7 (0.49) | 2 (0.69) | 9 (0.52) |  |  |  |  |  |  |  |  |
| ***15:03g*** | 83 (5.76) | 6 (2.08) | 89 (5.15) |  |  |  |  |  |  |  |  |
| ***16:01g*** | 11 (0.76) | 3 (1.04) | 14 (0.81) |  |  |  |  |  |  |  |  |
| ***16:02g*** | 25 (1.74) | 1 (0.35) | 26 (1.5) |  |  |  |  |  |  |  |  |
| **Total** | 1440 | 288 | 1728 | **Total** | 1440 | 288 | 1728 | **Total** | 859 | 170 |  |
